# Development of an Anatomically Realistic Forward Solver for Thoracic Electrical Impedance Tomography

**DOI:** 10.1155/2013/983938

**Published:** 2013-03-24

**Authors:** Fei Yang, Jie Zhang, Robert Patterson

**Affiliations:** ^1^Washington University School of Medicine, Saint Louis, MO 63110, USA; ^2^Division of Radiological Medical Physics, University of Kentucky, Lexington, KY 40536, USA; ^3^Institute of Engineering in Medicine, University of Minnesota, Minneapolis, MN 55455, USA

## Abstract

Electrical impedance tomography (EIT) has the potential to provide a low cost and safe imaging modality for clinically monitoring patients being treated with mechanical ventilation. Variations in reconstruction algorithms at different clinical settings, however, make interpretation of regional ventilation across institutions difficult, presenting the need for a unified algorithm for thoracic EIT reconstruction. Development of such a consensual reconstruction algorithm necessitates a forward model capable of predicting surface impedance measurements as well as electric fields in the interior of the modeled thoracic volume. In this paper, we present an anatomically realistic forward solver for thoracic EIT that was built based on high resolution MR image data of a representative adult. Accuracy assessment of the developed forward solver in predicting surface impedance measurements by comparing the predicted and observed impedance measurements shows that the relative error is within the order of 5%, demonstrating the ability of the presented forward solver in generating high-fidelity surface thoracic impedance data for thoracic EIT algorithm development and evaluation.

## 1. Introduction

Electrical impedance tomography (EIT) is a medical imaging technique in which an image of the conductivity distribution in a part of the body is inferred from surface electrical potentials resulting from application of a number of current patterns through the body. Because of its noninvasiveness, portability, and low cost, EIT has been actively investigated since the 1970s [1] and finds potential applications in a wide variety of clinical areas including monitoring of lung problems such as pulmonary edema [2] or pneumothorax [3], non-invasive monitoring of heart function and blood flow [4], localization of epileptic foci [5], investigating gastric emptying [6], and measuring local internal temperature increases associated with hyperthermia therapy [7]. Lately, similar technique has also been proposed for stenotic plaque detection [8].

Among those, one of the most promising applications of EIT is continuous regional pulmonary monitoring, especially for monitoring patients being treated with mechanical ventilation. Mechanical ventilation is indicated when the patient's spontaneous ventilation is inadequate and is one of the most common interventions administered in intensive care. Mechanical ventilation can improve the prognosis for acute phase patients; however, it also often leads to potential complications such as ventilator-associated lung injury (VALI) and ventilator-induced lung injury (VILI). It thus poses an urgent demand on continuous and noninvasive monitoring of regional ventilation at the bedside of patients with respiratory failure. The feasibility of EIT for monitoring regional ventilation as demonstrated by previous studies on the one hand and the capability of EIT to resolve the volume changes between dependent and nondependent lung regions as ventilator parameters change thus allowing patient-specified ventilator settings during lung protective ventilation on the other have fostered a growing interest in using EIT for monitoring patients under mechanical ventilation [9].

However, in most clinical or research settings images are reconstructed using a variety of algorithms and thus show spatial nonuniformity in image amplitude, position, and resolution, thereby making interpretation of regional ventilation difficult or even error prone [10]. In addition, these algorithms have poorly understood behaviors in real patients, which makes it difficult to interpret whether a certain behavior is real or artefact due to the algorithms. As an initiative to address this issue, GREIT (Graz consensus Reconstruction algorithm for EIT) was proposed in an attempt to develop a consensus framework for a reconstruction algorithm [10]. One of the major challenges for developing such a reconstruction algorithm is the dearth of well-accepted standard thoracic impedance datasets. This is due to the costs of acquisition and the difficulties of *in vivo* quantifying the severity of lung lesions as well as the ethical issues in sharing patient data amongst others. In view of this need, this paper presents an anatomically detailed forward solver for thoracic EIT built based on ECG-gated MR images from a representative adult male aiming at generating well-characterized thoracic impedance datasets for thoracic EIT reconstruction algorithm development and evaluation.

## 2. Methods

### 2.1. The Electrical Model of the Thorax

**Figure 1:**
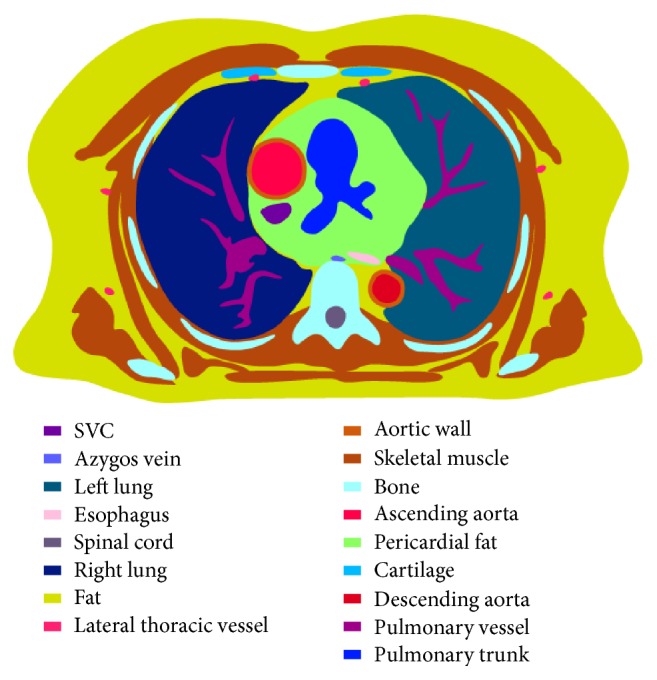
Sample of a segmented axial image. Inferior view at T6 vertebral level.

The subject imaged as the anatomical source for the model was a 63-year-old male, weighing approximately 100 kg and approximately 180 cm in height. MR scans were performed with a 1.5 T Siemens Sonata instrument. Forty-three breath-held thoracic transverse images, gated to coincide with end diastole, were obtained from abdomen to neck. The images were digitized with transverse resolution of 1.5 mm × 1.5 mm and axial resolution of 5 mm (equal to the MR slice thickness). Organs and tissues were segmented manually and confirmed by a pathologist. Upon segmentation, a total of thirty-six tissue types and blood-containing regions were obtained. For each of the identified components, a 3D volumetric mask was created in the interest of automatic electrical conductivity assignment. Conductivities for the major tissues used in the model are listed in Table 1 [11]. A 3D electrical model of the thorax at the end of diastole was thus created, with a resolution of 1.5 × 1.5 × 5 mm^3^ and 3.8 million elements. As a sample, one segmented slice is shown in Figure 1.

### 2.2. The Forward Problem

In the electrical frequency range of 1 kHz to 1 MHz, the human body can be conceived of as a piecewise, homogeneous, and resistive system with neglecting the reactive effects of the body [12]. If it is also assumed that the thorax is a source-free region and that the current flux normal to the body surface is zero except under the current electrodes, then for a given conductivity distribution in the defined volume conductor, the potential distribution induced by current sources obeys the generalized Laplace's equation subject to the integrated Neumann boundary conditions on the electrodes and the Dirichlet boundary conditions on other body surface. Due to its complex geometry and inhomogeneities in electrical conductivity, the modeled thoracic volume was discretized into hexahedral elements and the finite difference method was employed for the numerical solution of the governing equation. By applying Ohm's and Kirchoff's laws on the discretized nodes, equations describing the potential at one node as a function of the potentials at the adjacent nodes were established, from which a large sparse system of linear equations accounting for the potentials throughout the modeled conductive volume was able to be assembled and solved consequently.

### 2.3. The Graphic User Interface Environment

The above-mentioned modeling approach requires many steps and involves multiple applications. To achieve ease of use, model data and simulation programs were further integrated in a MATLAB-based Graphic User Interface (GUI) environment that guides users through the simulation process in a step-by-step manner. The following lists some important features implemented in the environment.

**Figure 2:**
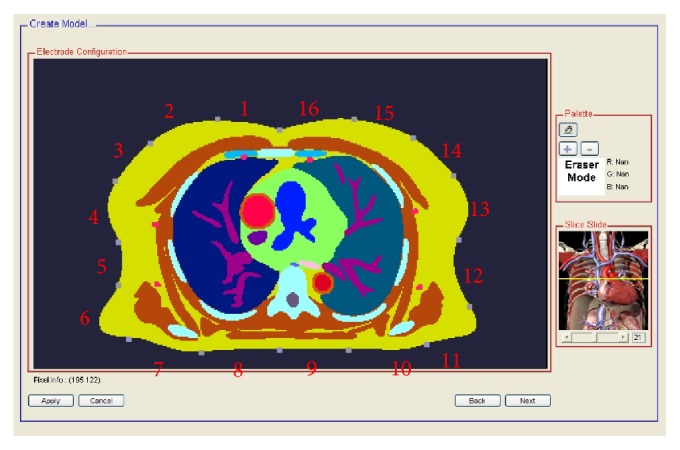
A screen shot of the developed GUI. Electrodes are paired and numbered to show the default adjacent excitation scheme.

(1) Ability to modify the accompanying thoracic model, if necessary. The segmented anatomical geometry can be changed in a pixelwise fashion and/or as a whole be linearly scaled up or scaled down up to 20%. Furthermore, electrical conductivities are tabularized, allowing adjustment of the conductivity for any given tissue.
(2) Interactive electrode placement. The GUI allows users to interactively insert electrodes into the rendered images of the segmented volume. By default, there are 16 electrodes evenly spaced around the body periphery at a mid-thoracic level and the adjacent excitation scheme is used.  Figure 2 shows a screen capture shot of the interface screen for electrode placement and the assumed electrode pairs for the adjacent excitation strategy.
(3) Automated electrode arrangement recognition. The developed software environment is able to automatically cluster the placed dot electrodes into distinct groups based on spatial adjacency. Upon recognition, the electrodes are paired and numbered anticlockwise starting with electrode pair 1 at the 12 o'clock position.

Upon completion of simulations, the software generates two output files. One is a picture file which gives the location of each electrode pair, and the other gives the transfer impedance data which can be readily used or can be easily adapted to the expected form of a specific algorithm, for image reconstructions.

### 2.4. Image Reconstruction

The reconstruction of conductivity distribution from surface voltage measurements is complicated by the fact that electric current is distributed over the entire volume being modeled. Therefore, reconstructing the electrical conductivity distribution of the volume conductor is significantly more difficult than that of other medical imaging modalities such as CT where the photon travels essentially in straight lines. Besides that, the ill-conditioned nature of problems of this kind imposes further difficulties. Forty years after the first impedance image was published, EIT reconstruction continues to be an area of active research. Among various proposed reconstruction algorithms [13], two most representative algorithms, the Sheffield filtered back-projection algorithm and the GREIT algorithm [10], were taken as examples in the current study to show the utility of the developed software environment as a forward solver for thoracic EIT. The Sheffield filtered backprojection algorithm, distributed in the commonly used Sheffield DAS-01 P EIT system, aims at projecting changes of the surface impedance measurements along the equipotential lines calculated from the homogeneous medium. The GREIT algorithm, developed by a consensus of a large group of experts in EIT algorithm design and clinical applications for pulmonary monitoring attempting to provide a unified approach for real-time thoracic EIT reconstruction, derives the conductivity distribution based on a matrix pretrained with various performance requirement criteria such as uniform amplitude and uniform resolution. Current implementations of both algorithms reconstructed conductivity differential images onto a circular field of 32 × 32 pixel.

## 3. Results

### 3.1. Accuracy of the Forward Solution

Accuracy of the developed forward solver in predicting surface impedance measurements was appraised by comparing the predicted impedance measurements with the impedance measurements made on the same subject whose MR data was used as the anatomical source of the model. To obtain impedance measurements from the subject, 16 electrodes were placed evenly around the body plane of the subject as shown in Figure 2 and the impedance measurements were recorded with a BIOPAC system. For 16 electrodes with the adjacent excitation scheme, a total of 208 impedance measurements were obtained.  Table 2 presents the percentage of differences between the predicted impedance measurements and the observed impedance measurements. It shows that the relative error is within the order of 5%, demonstrating the ability of the presented forward solver in generating high-fidelity surface thoracic impedance data.

### 3.2. Reconstruction Demonstration

Impedance measurements generated from the developed forward solver for image reconstruction demonstration consisted of the following conductivity change scenarios of either or both lungs:

1. changing the conductivity of both lungs from the standard value of 0.0714 S/m up to 0.1000 S/m,
2. changing the conductivity of both lungs from 0.0714 S/m down to 0.0556 S/m,
3. changing the conductivity of the right lung from 0.0714 S/m up to 0.1000 S/m,
4. changing the conductivity of the right lung from 0.0714 S/m down to 0.0556 S/m.

**Figure 3:**
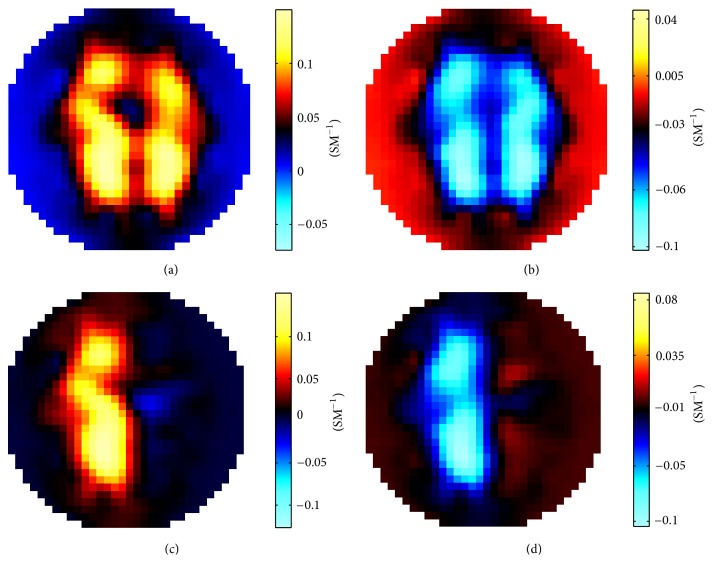
Conductivity differential images reconstructed with the Sheffield filtered backprojection algorithm. (a) Conductivities of both lungs were changed from 0.0714 S/m to 0.1000 S/m; (b) conductivities of both lungs were changed from 0.0714 S/m to 0.0556 S/m; (c) conductivity of the right lung was changed from 0.0714 S/m to 0.1000 S/m; (d) conductivity of the right lung was changed from 0.0714 S/m to 0.0556 S/m.

**Figure 4:**
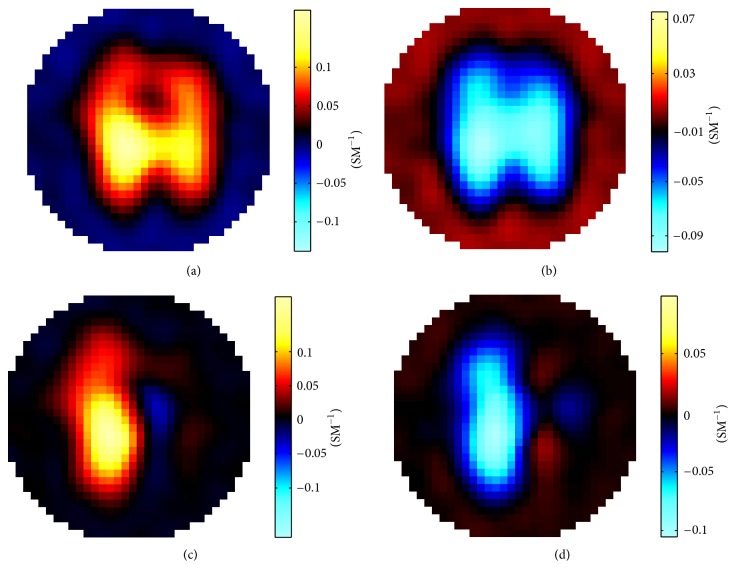
Conductivity differential images reconstructed with the GREIT algorithm. (a) Conductivities of both lungs were changed from 0.0714 S/m to 0.1000 S/m; (b) conductivities of both lungs were changed from 0.0714 S/m to 0.0556 S/m; (c) conductivity of the right lung was changed from 0.0714 S/m to 0.1000 S/m; (d) conductivity of the right lung was changed from 0.0714 S/m to 0.0556 S/m.

All the simulations were carried out on a Dell Precision T7400 workstation with a 2 GB memory and for each case it took about 4 hours. The differential images reconstructed with using the Sheffield filtered backprojection algorithm and the GREIT algorithm are presented in Figures 3 and 4, respectively. The reconstructed conductivity differences shown in these images exhibit location agreements with the lungs underwent conductivity change, demonstrating the utility of the developed forward solver in generating surface impedance measurements for thoracic EIT.

## 4. Discussion

The forward problem of EIT involves building a model to determine the surface impedance measurements when current excitation is applied on the boundary of the volume being modeled. Constructing such a model generally assumes the geometry and conductivity values are known and entails solving the governing Laplace's equation in the interior of the modeled volume with proper boundary conditions. In order to accurately determine surface voltage measurements as well as the interior electric fields, models with realistic representation of anatomical variability inside the thorax are highly desirable. To date, a number of electrical models of the human thorax have been developed. Based on the US National Library of Medicines Visible Human Man data, Kauppinen et al. [14] built a finite difference thoracic model that has a total number of control elements of 477,611 with size ranging from 0.011 to 0.78 cm^3^ and 28 identified tissue types. de Jongh et al. [15] developed a finite element thoracic model comprising 214,957 control elements and 5 identified tissue types. Aguel et al. [16] constructed a finite element model of the human thorax consisting of 933,409 control elements and 4 identified tissue types. Wtorek [17] developed a finite element model of the thorax that consisted of 18,165 control elements with size ranging from 0.053 to 20.83 cm^3^. Mocanu et al. [18] developed a thoracic model containing approximately 400,000 control elements with size of 0.054 cm^3^.

Compared to these existing thoracic electrical models, the thoracic model accompanying the developed forward solver comprising 36 different types of tissues and consisting of 3.8 million control elements—about three times more than the best model among those aforementioned—with a uniform element size of 0.011 cm^3^ represents the human thorax with a much higher level of anatomical accuracy, thus allowing more accurate prediction of surface impedance measurements and electric fields in the interior of the thorax. Furthermore, it is built based on MR scans of a living subject, thus offering a more authentic representation of thoracic anatomy in comparison with the majority of the existing models where cadaveric anatomy was employed in that most organs inside the thorax undergo structural and functional changes after death. Moreover, the presented forward solver, coming equipped with a user-friendly GUI and being independent from commercial software, achieves ease of use to a greater degree than the previously reported ones.

Given the ill-posed nature of EIT reconstructions, one may wonder how detailed a forward model needs to be, or even whether a model with fine details as presented in this work is needed. In our opinion, this is the line of reasoning which leads us to propose the use of highly accurate models. EIT is a low resolution modality, but for many applications, it is important to study whether a particular small feature can be seen in the images, and if so, whether its location in the images will be perturbed by nearby anatomical structures. In order to study this effect, it is important to have a thoracic model featured with a high level of anatomical accuracy. One limitation of the developed forward solver is the inaccurate representation of the electrode-body interface. For reconstruction approaches employing voltages measured from current injection electrodes, the electrode impedance would have an evident impact on the surface impedance measurements. However, for applications like the GREIT and Sheffield backprojection along with a number of other methods where current injection electrodes are not included in the voltage pick-up schemes, its effect on surface impedance measurements would be expected to be very limited as having been demonstrated here in the current study. Use of the presented thoracic forward solver would thus be restricted to reconstruction strategies leaving out injection electrodes for impedance measuring. Incorporation of more realistic electrode models into the developed forward solver is currently under investigation.

## 5. Conclusion

In summary, we have developed an anatomically detailed forward solver for thoracic EIT equipped with a user-friendly GUI. We hope and expect that this software environment will be able to serve as a source to generate impedance measurements along with electric field data in aid of development and evaluation of thoracic EIT reconstruction algorithms.

## Figures and Tables

**Table 1:** Conductivity values of the major tissues.

Tissue	Conductivity (S/m)
Air	10^−18^
Lung	0.0714
Blood	0.6667
Heart muscle	0.4
Liver and kidney	0.1667
Skeletal muscle	0.4444
Fat, bone, and cartilage	0.05

**Table 2:** Percentage of difference between the predicted and observed impedance measurements.

		Voltage pick-up electrode pair															
		1	2	3	4	5	6	7	8	9	10	11	12	13	14	15	16
Current excitation electrode pair	1		2.2	−2.1	−3.9	1.5	−0.7	2.7	2.4	1.0	−2.8	−1.5	0.1	−1.2	−4.0		
	2			−2.5	−4.6	−3.3	0.3	−4.1	−3.0	4.2	1.1	−2.3	0.6	−0.9	−4.8	2.4	
	3	3.7				−0.5	−0.9	4.9	3.5	0.7	2.6	2.6	−1.5	0.5	2.9	0.6	−3.0
	4	−4.1	−4.6				−0.5	1.6	−3.3	−4.1	3.8	1.0	0.5	1.9	2.0	3.9	4.7
	5	−1.3	−1.6	−1.9				3.4	−0.4	3.2	3.2	4.0	−0.5	−0.1	0.3	2.4	−4.3
	6	4.0	4.6	1.8	3.4				−5.0	2.4	2.6	3.7	2.0	−0.1	2.7	0.9	−2.3
	7	2.2	−3.5	−4.5	3.8	−3.4				3.5	0.8	−4.4	−2.4	−3.2	−3.4	−0.9	0.1
	8	−0.5	−4.3	4.7	0.0	2.5	−2.5				4.5	−4.6	3.1	−2.9	3.1	2.4	−0.8
	9	−4.6	−1.8	0.6	−4.6	−3.1	1.2	3.3				−0.8	−1.9	−1.4	2.7	−1.0	2.6
	10	−4.6	−2.6	1.2	1.6	2.6	−0.7	−1.0	−4.6				3.2	1.2	3.3	−2.1	0.4
	11	3.4	−2.4	−2.7	−0.9	−4.3	4.2	2.4	5.0	−0.5				−3.1	2.7	2.8	−4.6
	12	−4.7	1.1	−3.0	2.8	1.5	4.5	−1.5	−2.7	3.6	0.1				0.6	3.8	−0.5
	13	−4.6	−1.6	3.1	−2.5	3.0	−0.3	−1.9	−3.2	−3.5	1.6	−3.2				2.0	−0.2
	14	0.1	3.3	0.1	2.4	2.5	−2.8	−2.5	−3.7	−1.2	−4.0	4.8	4.2				2.7
	15	−3.0	−2.1	0.5	−0.1	−1.2	−4.3	0.5	4.2	1.5	1.3	4.6	−4.3	−3.8			
	16		4.7	−1.5	−2.0	0.3	3.7	4.2	1.0	−0.1	3.9	3.3	−2.8	−0.5	3.2		
